# Herlyn-Werner-Wunderlich Syndrome: A Case Report

**DOI:** 10.31729/jnma.8376

**Published:** 2023-12-31

**Authors:** Deepak Lamichhane, Anshu Sutihar, Gubeanthrey Janakyraman, Rudish Jaz Shrestha, Mahbubur Rahman Razeeb

**Affiliations:** 1 Dhaka Medical College, Bakshi Bazar, Dhaka, Bangladesh; 2 Department of Obstetrics and Gynaecology, Dhaka Medical College, Bakshi Bazar, Dhaka, Bangladesh

**Keywords:** *case reports*, *dysmenorrhea*, *uterine didelphys*

## Abstract

Herlyn-Werner-Wunderlich syndrome is a rare congenital malformation of the Mullerian ducts characterized by uterine didelphys with obstructed hemivagina and ipsilateral renal agenesis. Commonly, such patients present with pelvic pain, dysmenorrhea following menarche, and an abdominal mass secondary to hematometrocolpos. In this report, a case of a 14-year-old female presented with abdominal pain, back pain and acute urinary retention. She attained menarche at the age of 10 years; however, symptoms of dysmenorrhea only appeared 4 years later. She was eventually diagnosed with the help of ultrasound and computed tomography urogram. She was managed conservatively with an incision and drainage procedure and was also advised for resection of vaginal septum. The nonspecific nature of symptoms such as regular menstruation with cyclical abdominal pain impedes the diagnosis which can lead to an array of complications. Awareness of this syndrome can help avoid misdiagnosis and allow for early surgical intervention.

## INTRODUCTION

Herlyn-Werner-Wunderlich (HWW) syndrome is defined as a rare form of Mullerian duct anomalies characterized by a triad of obstructed hemivagina, uterine didelphys, and ipsilateral renal agenesis.^1^ This case was first reported by Purslow in 1922 and was later described by Herlyn and Werner in 1971.^2,3^ This is a rare congenital syndrome with the reported incidence of 0.1% to 3% in the general population.^4^ Common presenting symptoms include increasing pelvic pain and dysmenorrhea following menarche.^5^ In our case the symptoms appeared four years after menarche instead of following the typical pattern of symptom presentation soon after achieving menarche.

## CASE REPORT

A 14-year-old female presented to the emergency and trauma department with complaints of severe lower abdominal and back pain for the past 3 months. Characteristic lower abdominal pain which was colicky in nature, radiating to the back, and often associated with nausea and vomiting would begin with the onset of her menses and persist throughout her menstrual cycle which typically lasted for 5 to 7 days. In addition to that, she also had experienced symptoms consistent with urinary retention 24 hours prior to admission. She attained menarche at the age of 10 years and experienced a regular menstrual cycle every 28 days with scanty flow. The pregnancy was unremarkable and she was born at term with no family history of congenital anomalies. She had not experienced any other health ailments since birth. She was neither sexually active nor taking any form of contraceptive.

A few months before admission, she began to experience severe dysmenorrhea, which prompted her caretakers to consult a private general practitioner; however, there was no definite diagnosis. She was symptomatically treated with an antispasmodic agent and analgesic, which improved her symptoms briefly. She was then referred to a tertiary care centre for further workup, management, and continuation of care.

**Figure 1:**
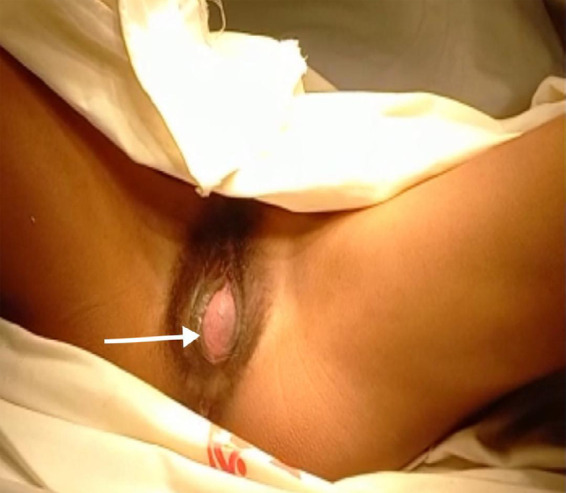
Obstructed hemivagina with bulging hymen.

General examination revealed mild anaemia and an abdominal examination revealed a tense urinary bladder. Per vaginal examination revealed a bulged hymen with obstructed hemivagina (Figure 1).

**Figure 2:**
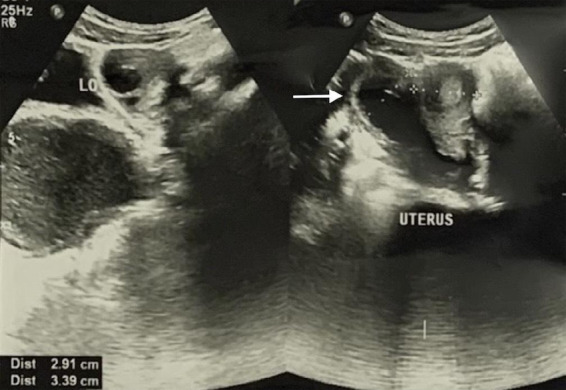
Ultrasound showing uterine didelphys.

She was afebrile and her vital signs were within normal limits. Routine investigations such as a complete blood count and urine routine microscopic examination (RME) were done. The patient's complete blood count revealed a haemoglobin level of 11.5 g/dl, white blood cell count was 6.07 x 103/mm^3^ with normal differential counts. Her platelet count was 259 x 103/mm^3^. Urine RME revealed no signs of infection; pus cells: 1-2/HPF; epithelial cells: 2-4/HPF; RBC: nil. Further evaluation with ultrasonography of the whole abdomen revealed the uterus to have a separate fundus, body, cervix, and vagina with mild to moderately dense endometrial collections in the right moiety of the didelphys uterus which is continuous with the large dense collection (12.5 x 6 cm) within the vagina (Figure 2).

**Figure 3:**
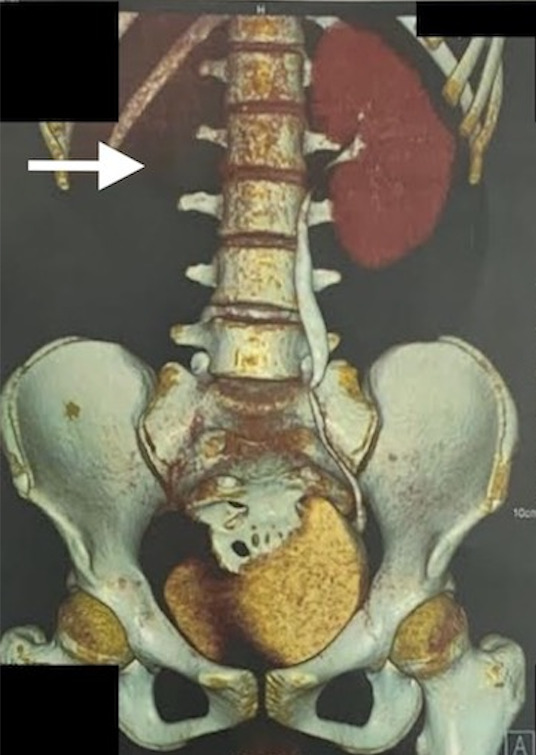
CT urogram with 3D reconstruction showing absent right kidney and ureter.

The left moiety of the uterus showed normal features. A non-visualized right kidney (agenesis) was also appreciated via ultrasonography. A diagnosis of didelphys uterus with hematometrocolpos with an absent right kidney was concluded. Additional imaging with a computed tomography (CT) urogram was performed, and the CT urogram report revealed an absent right kidney and ureter, along with uterine didelphys with hematocolpos and a normally excreting left kidney (Figure 3).

Magnetic resonance imaging (MRI) was not done due to the patient's financial constraints. The patient was conservatively managed for hematocolpometra with an incision and drainage procedure. Characteristic black tarry blood was noted on drainage, which subsequently relieved symptoms of urinary retention, and pelvic pain gradually resolved. There were no preoperative or postoperative complications. The patient was then advised for surgical resection of the vaginal septum or vaginoplasty.

## DISCUSSION

HWW syndrome is defined as a triad of obstructed hemivagina, uterine didelphys, and ipsilateral renal agenesis, also commonly known as obstructed hemivagina and ipsilateral renal anomaly (OHVIRA) syndrome.^6^ Although the aetiology is not well established, it is thought to be a result of environmental and genetic insults to the development of paramesonephric ducts and metanephros.^4^ The overall incidence of uterine malformation among females is estimated to be 7 to 10%. Genital developmental malformations usually arise as a result of non-fusion or failed resorption of the uterine septum.^1^ Normally, the fallopian tubes, uterus, and upper two-thirds of the vagina develop from paramesonephric, and the lower one-third vagina from the urogenital sinus. According to vaginal morphology, HWW syndrome is classified as class 1 (completely obstructed hemivagina) and class 2 (incompletely obstructed hemivagina), both of which are divided into subclasses.^1,3,4^ Our patient presented with the classification class 1 and subclass 1.1 (with blind hemivagina).

The common presentation of the disease includes dysmenorrhea, increasing pelvic pain, and abdominal mass secondary to hematometrocolpos due to obstructed hemivagina. Urinary retention may result from tubo-ovarian abscess and pelvic inflammatory disease occurring as a result of infection of the clotted blood.^5^ Diagnosis is delayed in patients due to the non-appearance of symptoms until menarche, and those who present with complete hemivaginal obstruction have completely different symptoms from those who have incomplete hemivaginal obstruction.^2^ Normal menstruation from non-obstructed sites as well as treatment of dysmenorrhea with non-steroidal inflammatory drugs (NSAIDS) and oral contraceptive pills (OCPs) eliminate the menses and contribute to further delay in diagnosis.^3^ Hematometrocolpus develops after attaining menarche, and only after that do the patients become symptomatic.^7^ Sometimes patients can present with abnormal vaginal discharge, abdominal pain, vomiting, fever, infertility, complicated pregnancy and labour, as well as endometriosis.^1,2,6^ Our patient was also treated symptomatically with NSAIDS for pelvic pain, and the diagnosis was missed several times, possibly due to normal menstruation and the normal appearance of female external genitalia.

A correct diagnosis can be made using history and examination, along with appropriate imaging.^8^ The most widely used diagnostic tools are ultrasound and CT.^9^ Ultrasound is the first line of investigation that can identify renal agenesis, bicornuate uterus, and any collections in the vaginal or uterine cavity.^10^ MRI is a better investigation for soft tissue anatomy, which can delineate uterine and vaginal morphology, septum location, and other subtle findings. An MRI should be obtained prior to surgical planning and intervention.^10^ Laparoscopy is not a necessary investigation but can be used to confirm the diagnosis when it is not clear by imaging or when MRI is unavailable.^8^

Nowadays, the cornerstone of treatment is minimally invasive vaginoscopic surgery guided by the excision of the vaginal septum and drainage of the fluid.^10^ For symptomatic relief and to prevent pregnancy-related problems like miscarriage, preterm birth, malpresentation, low birth weight, and cesarean delivery, prompt identification and treatment of this syndrome is essential.^10^ Acute complications common to this syndrome include pyosalpinx and pyohematocolpos. Chronic pelvic pain, endometriosis, and pelvic adhesion brought on by retrograde menstruation are examples of remote consequences. Adenocarcinoma of the uterus and cervix and clear cell carcinoma of the obstructed uterus are two uncommon consequences that have been reported.^9^

HWW syndrome has a variable onset and mode of presentation, as demonstrated by our study, with the occurrence of symptoms only after 4 years following menarche, thereby not adhering to the predominant pattern of symptom presentation shortly after attaining menarche. Diagnosis can be easily overlooked due to the ambiguity of symptoms such as nonspecific cyclical menstrual pain and regular menstruation. We present this case to emphasize the necessity for early detection, which can be done even with simple imaging techniques that serve as excellent diagnostic measures, such as ultrasonography, followed by the employment of effective management. This is imperative for the prevention of complications and to preserve fertility. An MRI was not done due to the patient's financial constraints. Surgical incision of the blocked hemivaginal orifice and drainage of hematometrocolpos are the primary interventions that are necessary for the expedited relief of the symptoms experienced by the patient, which is then followed by a vaginal septum resection or vaginoplasty. A comprehensive treatment plan consists of a manifold process that includes the active role of a multidisciplinary team in order to avoid potential complications as well as to achieve a favourable prognosis.
